# Antigenic evidence of bluetongue virus from small ruminant population of two different geographical regions of Odisha, India

**DOI:** 10.14202/vetworld.2016.304-307

**Published:** 2016-03-21

**Authors:** Shaswati Subhadarsini Pany, Sanchay Kumar Biswas, Karam Chand, Nihar Nalini Mohanty, Laxmi Narayan Sarangi, Bimalendu Mondal, Hemant Kumar Panda

**Affiliations:** 1 Department of Veterinary Microbiology, Guru Angad Dev Veterinary & Animal Sciences University, Ludhiana - 141 004, Punjab, India; 2 Division of Virology, Indian Veterinary Research Institute, Mukteswar - 263 138, Uttarakhand, India; 3 Research and Development Laboratory, National Dairy Development Board, IIL Campus, Hyderabad, Telangana, India; 4 Indian Veterinary Research Institute, Kolkata Campus, Kolkata, West Bengal, India; 5 Department of Veterinary Microbiology, College of Veterinary Science & Animal Husbandry, Bhubaneswar, Odisha, India

**Keywords:** bluetongue, goat, Odisha, sheep

## Abstract

**Aim:**

The aim of the present study was to carry out antigenic detection of bluetongue virus (BTV) among the small ruminant population of two different geographical regions of Odisha (coastal and central) using recombinant VP7 (r-VP-7) based sandwich enzyme-linked immunosorbent assay (s-ELISA).

**Materials and Methods:**

Blood samples (n=274) were collected from two different geographical pockets of Odisha, which covered mostly the coastal and central regions. Of the total samples under study 185 were from goat and 89 were from sheep. The blood samples were tested for the presence of BTV antigen by r-VP7 based s-ELISA.

**Results:**

r-VP-7 s-ELISA detected BTV antigen in 52.43% and 44.94% of the goat and sheep population under study, respectively. This study highlights the antigenic persistence of BTV in the state for the 1^st^ time.

**Conclusion:**

This high antigenic presence in both sheep and goat population suggests an alarming BTV infection in field conditions which warrants more systematic study directed toward isolation and characterization studies as well as the implementation of control strategy for BT in Odisha.

## Introduction

Bluetongue (BT) is an arthropod-borne viral disease caused by BT virus (BTV) of the genus Orbivirus, and family *Reoviridae*. The virus primarily affects sheep and wild ruminants. Cattle serve as the most important reservoir host of BT [1,2] and the infection caused by it is mostly subclinical/in apparent. However, in recent past incidence of clinical disease by serotype 8 has also been reported among cattle population in Europe [3]. The infection is vectored by blood sucking *Culicoides* midges which abound in hot and humid climate thus leading to regional outbreak of the disease [3]. Of the 1400 species of *Culicoides* worldwide, 39 have been found in India. Very few species of *Culicoides* have been demonstrated to be vectors for BTV, with the principal vectors varying geographically. The state of Odisha is house to two such species of *viz*. *Culicoides circumscriptus* and *Culicoides huffi* but whether or not they play a role in disease transmission is unknown [4]. In India, the disease was first reported in the year 1964 in the state of Maharashtra [5] and of the 26 BTV serotypes circulating worldwide, 21 serotypes are reported to have blanketed across the geo-ecological regions of Indian subcontinent which were detected either on the basis of virus isolation or serology [6,7].

The State of Odisha is located in the eastern coast (17°49’- 22°36’N latitudes and 81°36’ -87°18’ E longitudes) of India and contributes significantly to the livestock population of the country with an estimated participation in census of around 2.43%, 4.82%, 6.09% of sheep, goat and cattle population, respectively [8]. Odisha is bordered by states of Andhra Pradesh, Chhattisgarh, Jharkhand, West Bengal, all of which have reported the presence of either the virus or antibodies against BTV [6,9,10]. The climate in Odisha is usually hot and humid thus harboring an environment conducive for the propagation of the vector population, which in turn could result in increased viral dynamics in the state. The presence of anti-BTV antibodies among the livestock population of the state have been shown in decade old studies and in recent past by Joardar *et al*. in 2014 [11] who highlighted the presence of the same among cattle, sheep and goat population of the four districts of eastern Odisha. However, no outbreaks of BT have been ever reported in Odisha and no attempts have been made to know the antigenic status of animals for BT in the state. This lack of antigenic data among the small ruminant population of the state is a blind spot in the vision of surveillance of BT infection thus limiting the knowledge of viral dynamics in the state.

Keeping in mind the above necessities this pilot study was carried out for detection of BTV antigen among the sheep and goat population of Odisha. The study was the first of its kind to be performed in the state and provides a glimpse of viral activity among the susceptible small ruminant population.

## Materials and Methods

### Ethical approval

This study was conducted after approval by the Research Committee and Institutional Animal Ethics Committee.

### Blood samples

A total of 274 blood samples were collected between October 2011 and March 2012, at random from coastal and central regions of Odisha (Ganjam, Dhenkanal, Cuttack and Sambalpur districts) out of which 185 were of goat and 89 were of sheep. The clotting of blood samples was prevented by use of heparin (10 IU/ml) and was stored at 4°C until further use.

### Sandwich enzyme-linked immunosorbent assay (s-ELISA)

Group specific VP7 based s-ELISA was carried out as per the methodology of Chand *et al*. [12]. Briefly, capture antibody (rabbit hyperimmune serum (HIS), against purified whole BTV) diluted in carbonate bicarbonate buffer, pH 9.6 (Sigma, St. Louis, MO, USA) was coated onto wells of ELISA plate (Maxisorp) for 1 h at 37°C or 4°C overnight. Plate was washed 3 times with washing buffer (phosphate buffered saline [PBS] plus 0.05% Tween 20). Blocking was done with blocking buffer (4% skimmed milk in PBS) at 37°C for 1 h. After washing, control and test antigens were added to the plate and incubated. In an ELISA plate, four wells of each control, namely, positive antigen control, negative (uninfected baby hamster kidney cell culture supernatant) antigen control, conjugate control (no detection antibody) and blank (blocking buffer) control were incorporated. After further washing, detection antibody (Guinea pig HIS against BTV core particles) was added to the plate and color reaction was developed by adding a conjugate-substrate solution which was read on an ELISA plate reader at 492 nm.

### Interpretation of results

A sample was classified as positive when the optical density (OD) was equal to or greater than twice the OD of negative control (P/N ≥2) [12].

## Results and Discussion

The state of Odisha is broadly divided into four geographical or physiographic regions, i.e. Northern Plateau, Central Table Land, Eastern Hills, and Coastal Plains. The Coastal Plains form an extensive alluvial tract lying between the Eastern Ghat hill ranges and the coast and include parts of Balasore, Cuttack, Puri, and Ganjam districts. The Central River Basin occurs between the Northern Plateau and the Eastern Ghat hill ranges and covers parts of Bolangir, Sambalpur, Dhenkanal and Cuttack districts [13]. The present study included samples from the districts of Ganjam, Dhenkanal, Cuttack and Sambalpur representing two different geographical regions of Odisha. The climate in the candidate regions of the state is hot and humid for most parts of the year [13], thus predisposing the livestock populations to the threats of vector mediated viral infection. In the present study, an attempt was made for demonstration of BTV antigen from blood samples of the small ruminant population of the region under study using a polyclonal antigen based s-ELISA using anti rVP7 antiserum. The percentage of animals tested positive for antigen in the case of goats was 98 (52.43%) whereas in the case of sheep 40 (44.94%) samples were found to be positive.

VP7 is a viral core protein that is conserved within each serogroup and polyclonal and monoclonal antibodies raised to core particles may be used as a capture antibody in s-ELISA for detection of viral antigen in blood directly [3]. The test could detect virus as early as from 5 to 35 days post infection. Rabbit antiserum to purified BTV particles and guinea-pig antiserum to core particles were used as capture antibody and detection antibody respectively. Thereby our study runs parallel with that of Mondal *et al*. [14] who used a polyclonal antibody-based s-ELISA [11] for detecting BTV in the blood of goats which showed a mixed infection to peste des petits ruminants virus (PPRV). Similarly, in a different approach Gandhale *et al*. [15] characterized a monoclonal antibody (MAb) specific for the BTV group specific antigen (VP7) and used it to develop a s-ELISA for rapid diagnosis of acute BT, however, the author found 29.42% of the randomly collected field samples to be positive in the test. An MAb-based ELISA was also developed by Yin *et al*. [16] for the detection of BTV from cell culture lysates and blood samples of sheep.

In our study, out of the 52.43% goats samples found positive in s-ELISA, 9.32% of animals were strongly positive suggesting active infection whereas the remaining weak positive samples could be indicative of very recent infection. Similarly, in the case of sheep samples 11.43% out of the positive ones were marked as strong positive. The districts of Dhenkanal showed the highest apparent prevalence of BTV antigen among goats (70%), whereas in case of sheep samples Ganjam district was marked out of the others with an apparent antigenic prevalence of 50%. However, sampling bias and animal distribution in the region cannot be overruled for the disparity in results.

It was observed in the study that goats showed more antigenic prevalence of BTV in comparison to sheep, the reason of which cannot be fully ascertained although a number of hypotheses can be put forth. The high prevalence of BT in goats may be because of immune-suppression due to predisposition of other viral diseases like PPR, foot-and-mouth disease and Goat pox [14]. In a similar study, the recombinant VP7 based s-ELISA was used to reveal the presence of BTV in blood samples of PPRV infected goats BTV [14]. As the disease remains in a subclinical state in goats whereas sheep exhibits clinical symptoms, so affected sheep may be either slaughtered or sold by the farmers thus resulting in lesser prevalence rate in sheep. Other reasons may be the persistence of viremic phase for a longer period in goats and cattle than sheep and chance of getting bitten with midges is more in goat and cattle than sheep (due to presence of wool). The higher prevalence of BT in goats has also been highlighted in the study carried out by Joardar *et al*. [11], among the small ruminant population of Odisha, who found a higher prevalence of anti BTV antibodies among goats (31.25%) than that of sheep (26.66%).

It was observed that the prevalence of infection took place in and around winters, in contrast to majority of reports which attribute period of monsoon to be the stage play of viral activity. Hence, the prevalence during the period of October 2011 to March 2012 may be accounted for possible cross border animal movement apart from responsible vector activity. In a similar observation Sreenivasulu *et al*. [6] highlighted BTV outbreaks during the north-east monsoon period (October to December) followed by the south-west monsoon period (June to September) in states like Tamil Nadu and Andhra Pradesh, respectively.

## Conclusion

BT hits the livestock industry with great economic losses due to death, abortions, weight loss or reduced milk yield and restriction on movement of live animals, their products. In such a scenario the presence of infection in both sheep and goat at such a high rate suggests an alarming situation which warrants more systematic study as well as implementation of control strategy for BT in Odisha. However, lack of isolation studies still overshadows the dynamics of the virus in the state and calls for further analysis of the vector virus relation the state. The presence of virus activity highlighted in our study is the first report from state and is well indicative of virus circulation under the nose of therapeutic, prophylactic and managemental strategies. Moreover, cross border animal movement and hassles in differential diagnosis adds up further to the relentless problem. Hence, a multi-dimensional strategy of proper diagnosis, treatment, and control is essential for combating the eminent threat of BT which overshadows the state.

## Authors’ Contributions

SSP and NNM collected samples and prepared the final draft of the manuscript. SKB, KC, and LNS designed the study. The study was carried out under the guidance of BM and HKP. All authors read and approved the final manuscript.
